# Designing a “Thinking System” to Reduce the Human Burden of Care Delivery

**DOI:** 10.5334/egems.299

**Published:** 2019-04-24

**Authors:** Gurvaneet S. Randhawa, Yan Xiao, Paul N. Gorman

**Affiliations:** 1 National Cancer Institute, US; 2 University of Texas at Arlington, US; 3 Oregon Health and Science University, US

**Keywords:** Care Coordination, sociotechnical system, Distributed Cognition, treatment burden, information technology, patient-centered, systems thinking

## Abstract

Cancer patients interact with clinicians who are distributed across locations and organizations. This makes it difficult to coordinate care and adds to the burden of cancer care delivery. Failures in care coordination can harm patients. The rapid growth in the number of cancer survivors and the increasing complexity of cancer care has kindled an interest in new care delivery models.

Information technology (IT) is an important component of care delivery. While IT can potentially enhance collaborative work among people distributed across locations, organizations and time, the current design and implementation of health IT adds to the human burden and often makes it a part of the problem instead of the solution.

A new paradigm is needed, therefore, to drive innovations that reframe health IT as an enabler (and a component) of a “thinking system,” in which patients, caregivers, and clinicians, even when distributed across locations and time, can collaborate to deliver high-quality care while decreasing the burden of care delivery. In a thinking system, the design of collaborative work in health care delivery is based on an understanding of complex interplay among social and technological components. We propose six core design properties for a thinking system: task coordination; information curation; creative and flexible organizing; establishing a common ground; continuity and connection; and co-production. A thinking system is needed to address the complexity of coordination, meet the rising expectation of personalized care, relieve the human burden in care delivery, and to deliver the best quality care that modern science can provide.

## Introduction

The rapid growth in the number of U.S. cancer survivors (projected to exceed 26 million by 2040) and the complexity of managing the effects of cancer and its treatments has led to the call for new care delivery models [1]. Cancer care delivery burdens patients and their clinicians who are distributed across space and organizations. We propose core design properties for leveraging the power of technology and new care delivery models to minimize this burden.

An early-stage lung cancer patient spends 1 in 3 days interacting with health care systems, receives care from 20 physicians, and manages 12 medications [2]. The shift of tasks from clinicians (e.g., medication management and wound care) to patients adds further to their treatment burden [3]. This treatment burden exacts a toll on the patient’s cognitive resources, time, finances and relationships, and contributes to the fatigue experienced by cancer patients [4,5,6]. One cancer survivor’s solution to improve care coordination was to become her own quarterback [7]. She created a detailed log of the physicians, their recommendations, her vital signs, and how she felt that day; some physicians found her log more useful than her official medical chart.

Coordination of cancer care also burdens clinicians. One primary care physician had 40 communications with other clinicians and 12 communications with the patient and caregiver over the course of 80 days of care for a cancer patient [8].

Care coordination failures can harm patients. For example, a patient with recently-completed treatment for acute myeloid leukemia was sent to the Emergency Department (ED) to receive a transfusion. He was not kept in isolation, developed a fever during transfusion, and was re-admitted to the hospital [9]. Three opportunities to coordinate this patient’s care were missed:

1. The existing care team didn’t communicate to the ED the need for patient isolation due to a history of neutropenia;
2. The differences in the electronic health record (EHR) interface between the ED and the oncology unit prevented access to relevant information from the oncology unit; and
3. The patient wasn’t informed of the significance of his neutropenia history.

This example illustrates the difficulty of providing continuity of care when clinicians are separated in time and space, are not supported optimally by information technology (IT) and fail to keep the patient in the loop.

Innovative care delivery models have been proposed to improve continuity of care for high-risk patients [10] but their reliance on a single physician is impractical to meet the needs of a patient’s journey across the cancer care continuum. The collaborative care model requires coordination between clinicians and is more effective than usual care in treating several chronic conditions; however, lack of understanding and buy-in of this model and poor communication processes and systems are barriers to its implementation in routine care [11].

IT is an important component of new care delivery models, including learning health systems [12]. While IT has the potential to enhance collaborative work among people distributed across locations, organizations and time, the current design and implementation of health IT makes it a part of the problem instead of the solution. Even experienced physicians who use advanced EHRs report a disruption of the patient interaction [13]. Current health IT increases the clinician’s effort to pull together information necessary for effective care coordination and adds to the care delivery burden.

## Thinking System

A new paradigm is needed, therefore, to drive innovations that reframe health IT as an enabler (and a component) of a “thinking system,” in which patients, caregivers, and clinicians, even when distributed across locations and time, can collaborate to deliver high-quality care while decreasing the burden of care delivery. In a thinking system, the design of collaborative work in health care delivery is based on an understanding of complex interplay among social and technological components; it is not designed to be a top-down system requiring a complete alignment of interests of all participants. This approach would seek to transform the existing collection of incoherent, disjointed activities into a cohesive system by synergistically using the capabilities of both humans and the IT systems. This approach is inclusive of, but is more than, the practice of systems-thinking for an organization and cognitive support for clinicians to practice evidence-based medicine. In this approach, recent advances in data science (“big data”) and artificial intelligence (AI) complement insights from research on collaborative work to reduce the burden of care delivery.

The thinking system concept is based on research on collaborative work that describes how tasks are performed by teams of people in different physical locations, in different organizations, and with differing backgrounds and dynamic goals. The theory of “Distributed Cognition” provides a framework for describing, measuring, and promoting goal-directed, information-rich, complex collaborative work [14, 15]. This framework, coupled with insights from the field of collaborative work, form the basis of our recommendation of designing a thinking system with six core properties:

- ***Task coordination.*** Each care delivery task takes place at the proper time, each actor fulfills their role, and all tasks are coordinated. A thinking system will organize resources to ensure the best path for care delivery is the easiest path: it will decrease the complexity of processes, create resilience to interruptions, transcend organizational boundaries, and continuously support individualized patient goals.
- ***Information curation.*** In a thinking system, individuals will have information they need, and use that information in ways that support collaboration and completion of patient care tasks. In the past, designers have often failed to appreciate the workload and barriers that health IT creates when patients and providers work to “curate” information needed to collaborate effectively: pulling data from disparate sources, validating and assessing provenance of data, organizing scattered information specific to a task, highlighting anomalous or critical information, and staging information to align with an activity flow. Such actions should be anticipated and supported, using automation and AI tools where this can reduce cognitive load.
- ***Creative and flexible organizing.*** Each patient possesses unique values and preferences, resources and constraints, and biological response to health conditions and interventions. A thinking system will help orchestrate an individualized care program that organizes distributed team members and resources and responds creatively to changes in patient goals and requirements. In a thinking system, technology will respond adaptively and productively as a patient’s journey unfolds.
- ***Establishing common ground.*** People in a thinking system will use IT and work processes to establish common ground, build and maintain shared expectations, and develop awareness of each other’s knowledge and assumptions.
- ***Continuity and Connection.*** The patients and their caregivers are often the only common thread connecting the disparate processes and organizations involved in their care. A thinking system, enabling people and processes of care, will transform care delivery from discrete, unconnected clinical encounters to coordinated and collaborative care that seamlessly connects individual encounters into a coherent process.
- ***Co-production.*** Patient-centered care is achieved only when patients and caregivers are active participants who co-produce care with their clinicians; the result is care that aligns with the patient’s goals and values. A thinking system will simplify the work of co-production, helping patients understand medical language and concepts and helping clinicians understand nuances in patient’s goals and preferences.

The goal of the thinking system is to reduce the human burden of delivering patient-centered care across diverse providers and care settings. Instead of asking individuals to adopt a systems-thinking approach, the thinking system is designed to support the individual tasks and coordination of activities to achieve the patient’s goals. A thinking system is needed to address the complexity of coordination, meet the rising expectation of personalized care, relieve the human burden in care delivery, and to deliver the best quality care that modern science can provide.
